# Supplementary figures and images for: Loss of Ptpmt1 limits mitochondrial utilization of carbohydrates and leads to muscle atrophy and heart failure in tissue-specific knockout mice
Source: eLife. 2023 Sep 6;12:RP86944. doi: 10.7554/eLife.86944 (PMC10482430; doi:10.7554/eLife.86944)

Whole blots of Figure 1-figure supplement 2

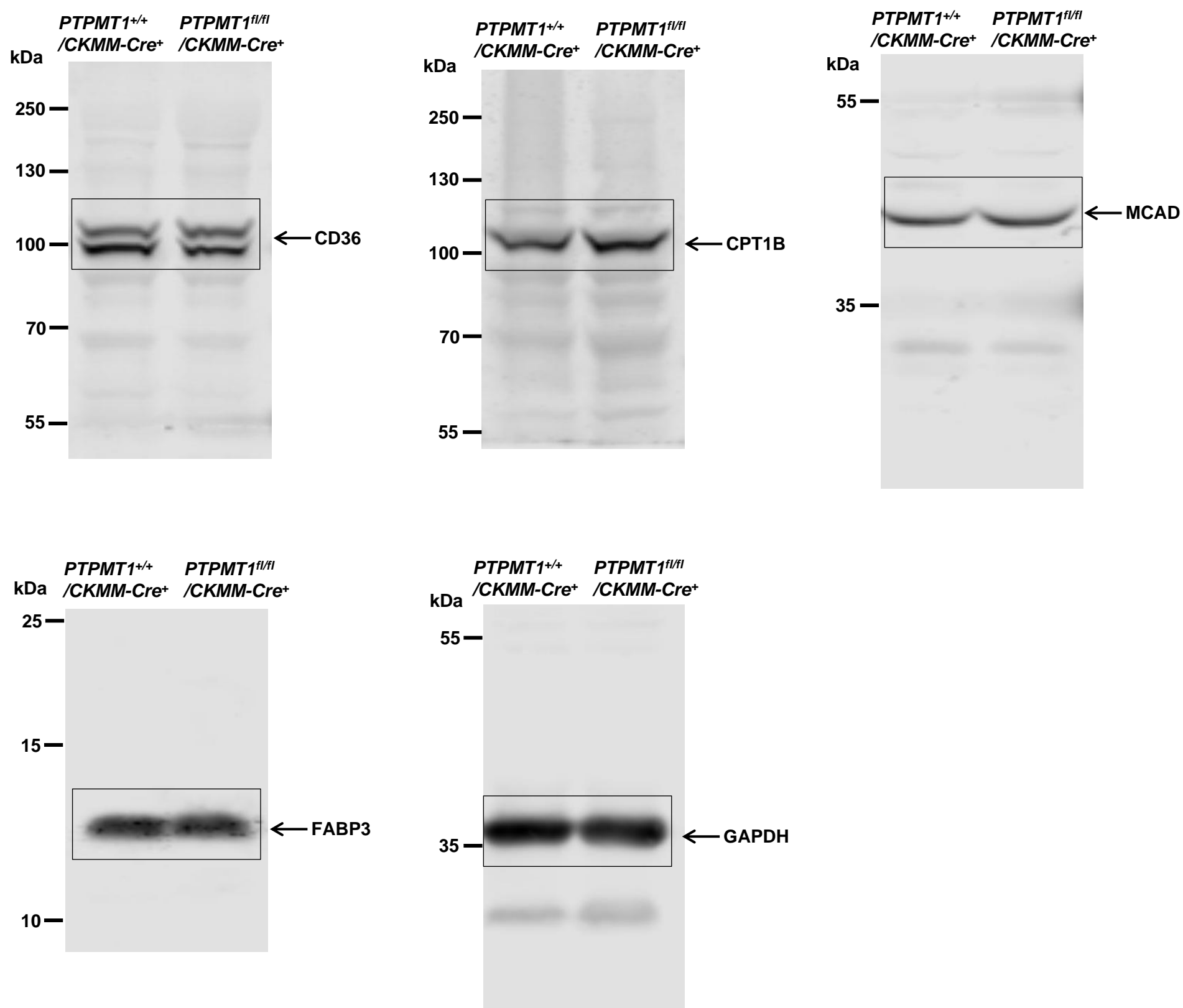

Supplement: Figure 1—figure supplement 2—source data 1. [file elife-86944-fig1-figsupp2-data1.pdf]

Whole blots of Figure 2

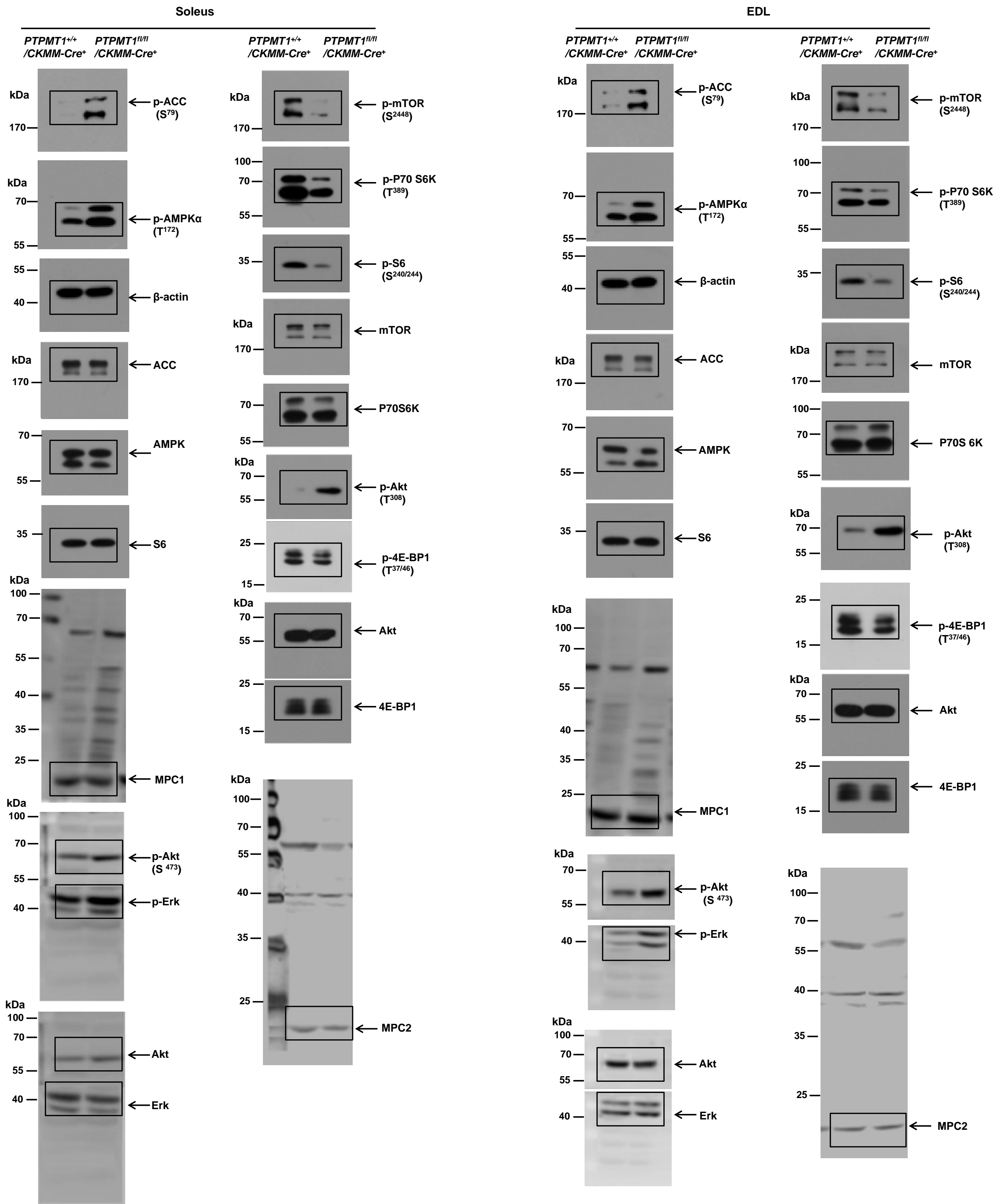

Supplement: Figure 2—source data 1. [file elife-86944-fig2-data1.pdf]

Whole blots of Figure 2-figure supplement 1

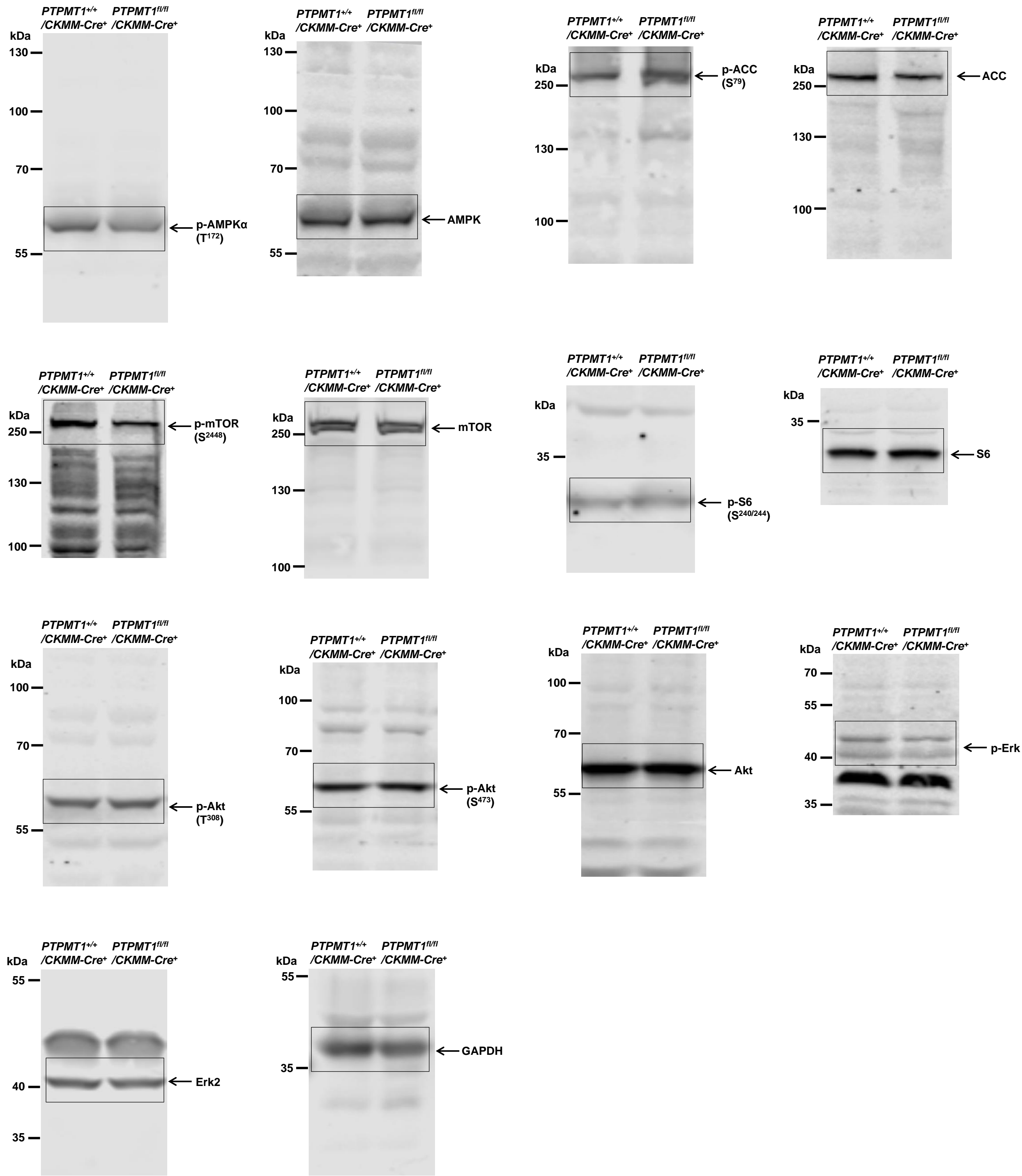

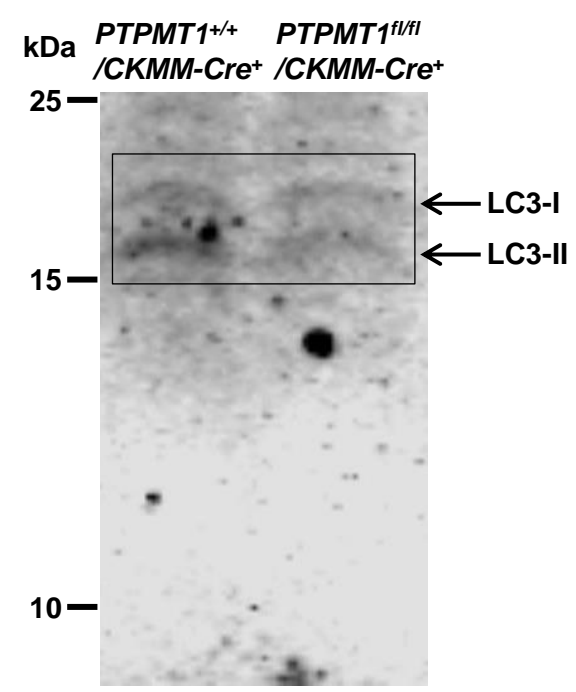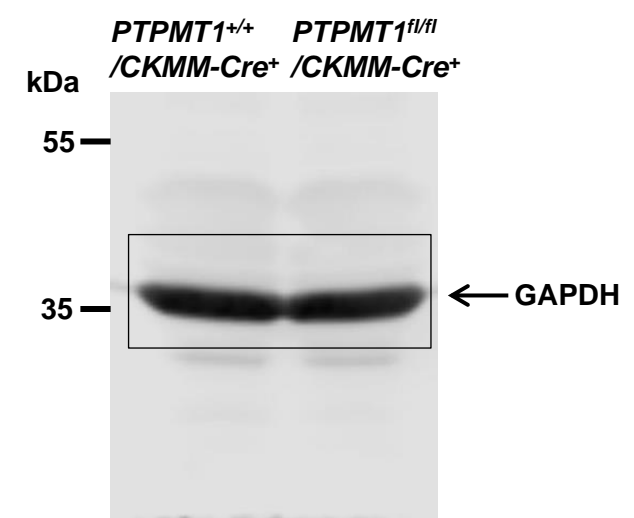

Supplement: Figure 2—figure supplement 1—source data 1. [file elife-86944-fig2-figsupp1-data1.pdf]

Whole blots of Figure 4-figure supplement 1

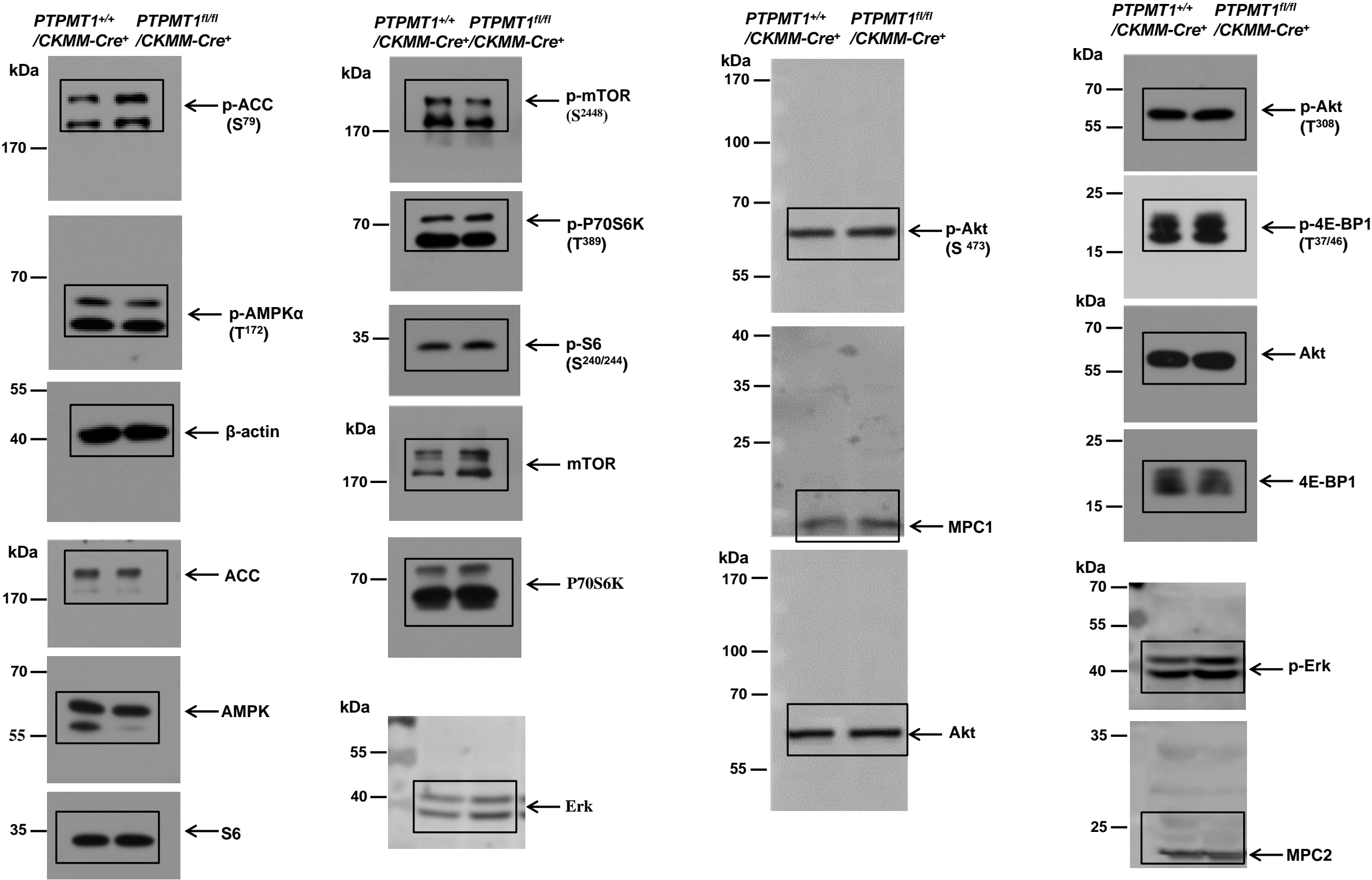

Supplement: Figure 4—figure supplement 1—source data 1. [file elife-86944-fig4-figsupp1-data1.pdf]

Whole blots of Figure 6

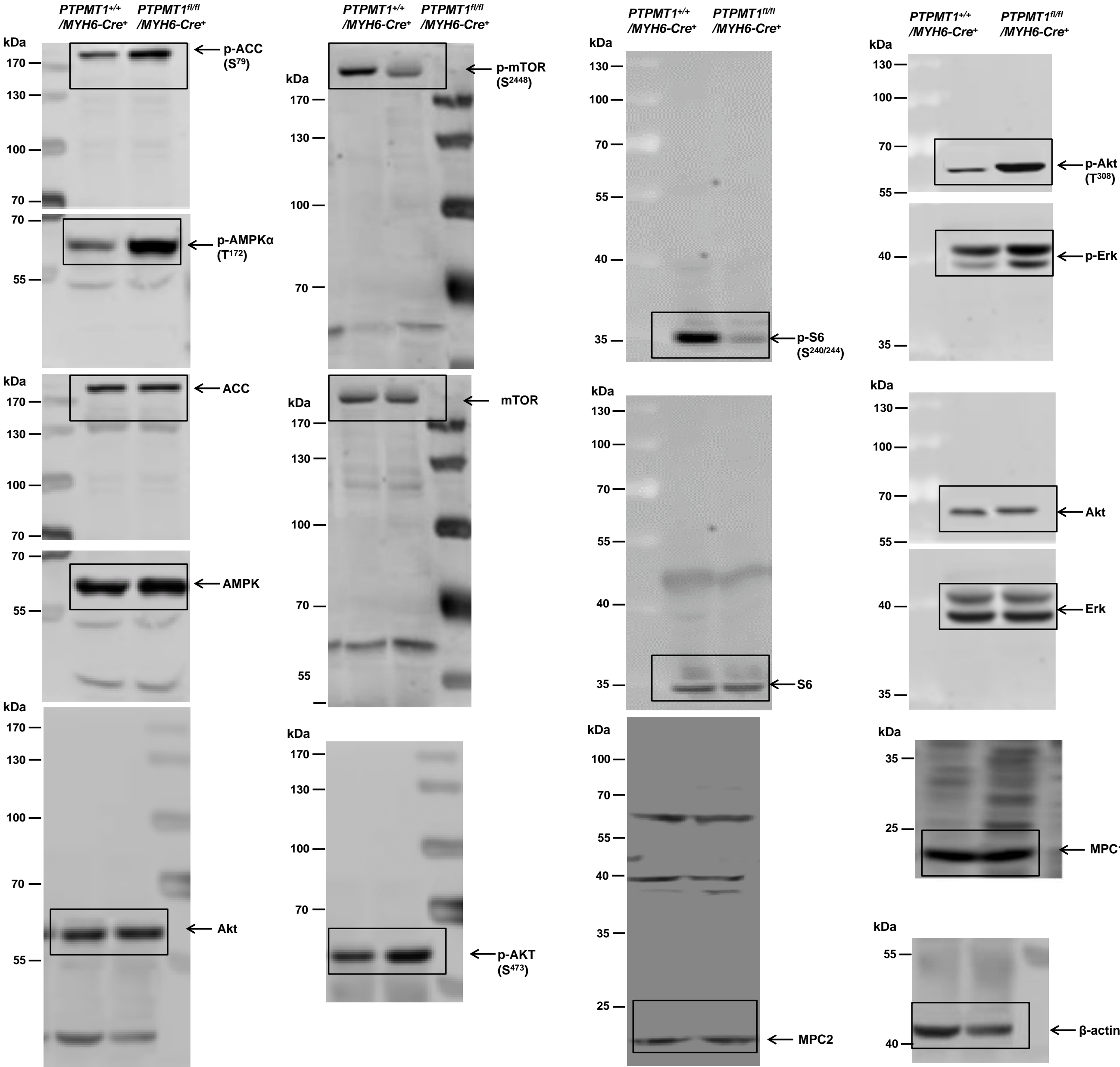

Supplement: Figure 6—source data 1. [file elife-86944-fig6-data1.pdf]

Whole blots of Figure 6-figure supplement 1

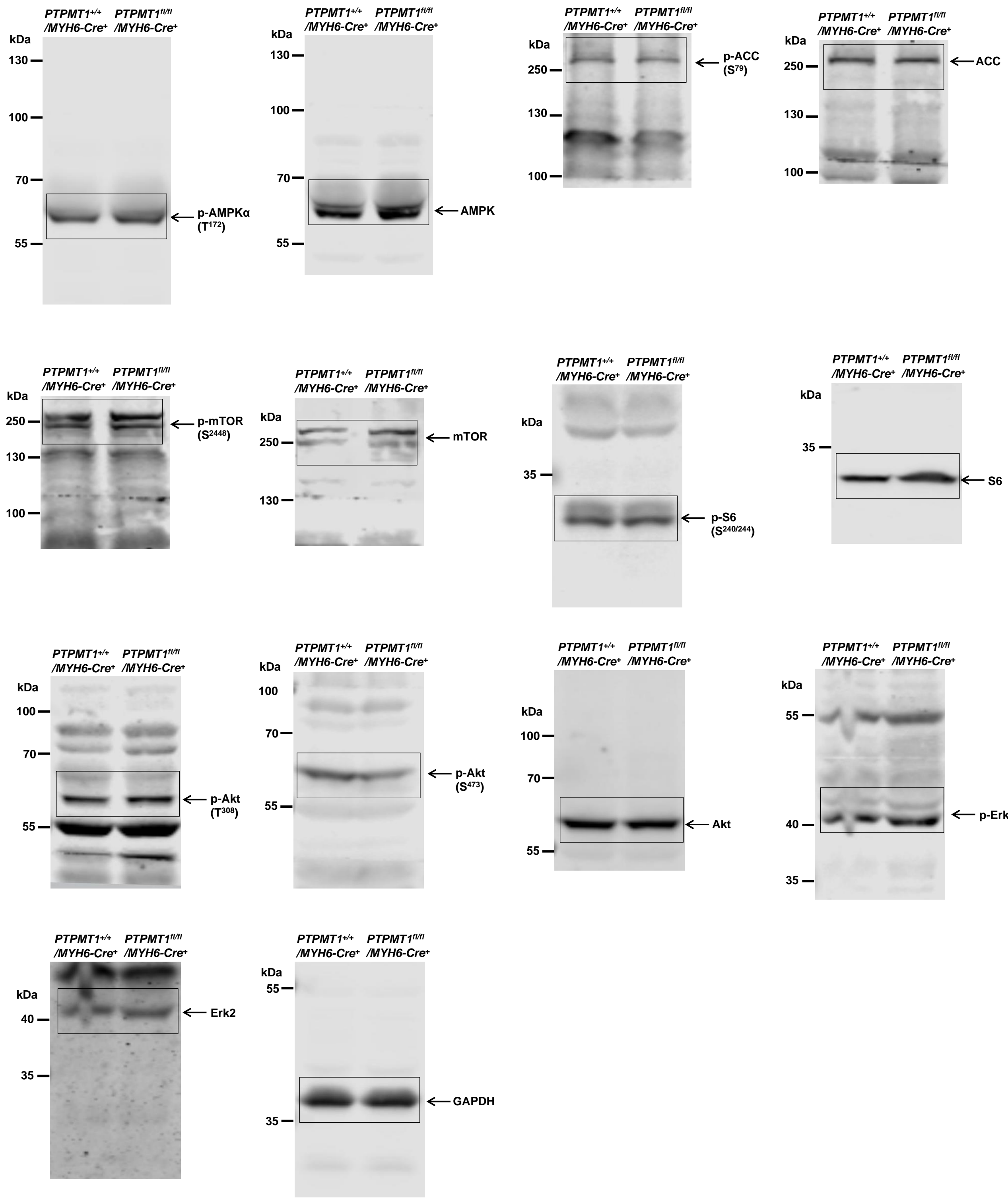

Supplement: Figure 6—figure supplement 1—source data 1. [file elife-86944-fig6-figsupp1-data1.pdf]
